# Proteolysis of the Human DNA Polymerase Delta Smallest Subunit p12 by μ-Calpain in Calcium-Triggered Apoptotic HeLa Cells

**DOI:** 10.1371/journal.pone.0093642

**Published:** 2014-04-01

**Authors:** Xiaoting Fan, Qian Zhang, Chao You, Yuanxia Qian, Jing Gao, Peng Liu, Huiqing Chen, Huifang Song, Yan Chen, Keping Chen, Yajing Zhou

**Affiliations:** 1 Institute of Life Sciences, Jiangsu University, Zhenjiang, Jiangsu, People's Republic of China; 2 School of Pharmacy, Jiangsu University, Zhenjiang, Jiangsu, People's Republic of China; University of Hawaii Cancer Center, United States of America

## Abstract

Degradation of p12 subunit of human DNA polymerase delta (Pol δ) that results in an interconversion between Pol δ4 and Pol δ3 forms plays a significant role in response to replication stress or genotoxic agents triggered DNA damage. Also, the p12 is readily degraded by human calpain *in vitro*. However, little has been done for the investigation of its degree of participation in any of the more common apoptosis. Here, we first report that the p12 subunit is a substrate of μ-calpain. In calcium-triggered apoptotic HeLa cells, the p12 is degraded at 12 hours post-induction (hpi), restored thereafter by 24 hpi, and then depleted again after 36 hpi in a time-dependent manner while the other three subunits are not affected. It suggests a dual function of Pol δ by its interconversion between Pol δ4 and Pol δ3 that is involved in a novel unknown apoptosis mechanism. The proteolysis of p12 could be efficiently blocked by both calpain inhibitor ALLN and proteasome inhibitor MG132. *In vitro* pull down and co-immunoprecipitation assays show that the μ-calpain binds to p12 through the interaction of μ-calpain with Pol δ other three subunits, not p12 itself, and PCNA, implying that the proteolysis of p12 by μ-calpain might be through a Pol δ4/PCNA complex. The p12 cleavage sites by μ-calpain are further determined as the location within a 16-amino acids peptide 28-43 by *in vitro* cleavage assays. Thus, the p12/Pol δ is a target as a nuclear substrate of μ-calpain in a calcium-triggered apoptosis and appears to be a potential marker in the study of the chemotherapy of cancer therapies.

## Introduction

Human DNA polymerase delta (Pol δ), a heterotetramer, is composed of the catalytic subunit p125 and three smaller subunits, p50, p68, and p12. It plays a crucial role in eukaryotic DNA replication and is also involved in various DNA repair processes and genetic recombination [1], [2], [3], [4], [5], [6].

Mammalian cells respond to DNA damage by a host of defense mechanisms, the DNA damage response (DDR). It involves the recruitment and assembly of large complexes of proteins that orchestrate and prioritize a network of responses that includes DNA repair, activation of cell cycle checkpoints and the decision for apoptosis. Current studies suggest that Pol δ is regulated in the DDR. The Pol δ smallest subunit p12 is degraded in response to DNA damage that is induced by UV irradiation, alkylating agents, oxidative, and replication stresses. This type of degradation leads to the consequent conversion of the holoenzyme Pol δ4 to the heterotrimer Pol δ3 lacking p12 [7]. Pol δ3 exhibits properties of an “antimutator” polymerase and is believed to contribute to an increased surveillance against mutagenesis [4], [8]. Analysis of the subcellular recruitment of Pol δ subunits to UV-damaged sites revealed that the Pol δ3 is the predominant form of Pol δ at the sites of UV damage as a result of p12 degradation. Pol δ3 was recruited to CPD damage sites in all phases of the cell cycle [9]. Therefore, the Pol δ3 is thought to be the primary form of Pol δ activity present after DNA damage likely involved in gap-filling reactions during DNA repair and also during translesion synthesis (TLS) in S-phase cells [5], [6], [9], [10]. In one of the proposed models for switching between TLS polymerases and Pol δ in DNA damage tolerance (DDT) pathway [11], [12], [13], the Pol δ3 is viewed as the primary form of Pol δ available for switching [1]. The Pol η/ub-PCNA/Pol δ3 complex is capable of switching rapidly between two states where Pol η and Pol δ3 can alternately occupy the primer terminus.

The degradation of p12 is thought to be dependent on an intact ubiquitination system under the control of ATR [7]. The UbcH5c and RNF8 are identified as an E2/E3 pair to be responsible for targeting p12 for its degradation. The p12 degradation regulated by RNF8 might be a major step in the assessment of Pol δ3 formation as a component of the cellular DNA damage responses [14]. More recently, the CRL4^Cdt2^, a key regulator of cell cycle progression that targets replication licensing factors, has been shown to target the p12 subunit in response to DNA damage and on entry into S phase [15]. It seems like that the CRL4^Cdt2^ could regulate the subunit composition of Pol δ during the cell cycle. However, if the DNA damage cannot be repaired in time in DDR, the consequent replication fork collapse, formation of aberrant fork structures, chromosome damage or loss, and the cell apoptosis will happen. The questions then arise as to what happened to p12 or other three subunits upon cell apoptosis. If the proteolysis of p12 does occur, how is the Pol δ complex sub-assembling(s) involved in and how to interconvert between Pol δ4 and Pol δ3?

Previous study has also shown that the p12 subunit might be one of the calpain targets. It is readily degraded by human μ-calpain in a reconstitution assay [16]. Calpain is an intracellular cysteine protease. It is activated by intracellular Ca^2+^ and cleaves and activates caspase-12 and cyclindependent kinase 5 (cdk5) to mediate calcium-triggered cell death [17], [18]. Therefore, besides its dependence on ubiquitination system, we believe that there must exist an alternative p12 degradation pathway that involves calpain and might play a role in calcium-triggered apoptosis. In this work, we demonstrate that the p12 is degraded in the early stage, restored thereafter, and then depleted again in the later stage in a time-dependent manner only in calcium-triggered apoptotic HeLa cells. The μ-calpain binds to p12 by the interaction of μ-calpain with other three subunit p125, p68, and p50, not p12 itself, as well as PCNA, which might be through a Pol δ4/PCNA complex. The p12 cleavage sites by μ-calpain are located within a peptide ^28^LAPELGEEPQPRDEEE^43^. The proteolysis of p12 could be efficiently blocked by both calpain inhibitor ALLN and proteasome inhibitor MG132. Thus, p12/Pol δ is a target as a nuclear substrate of μ-calpain. It appears that p12/Pol δ could be considered to be a potential marker in the study of the chemotherapy of cancer therapies.

## Results

### The smallest subunit p12 of Pol δ is degraded during a calcium-triggered apoptosis

**Figure 1:**
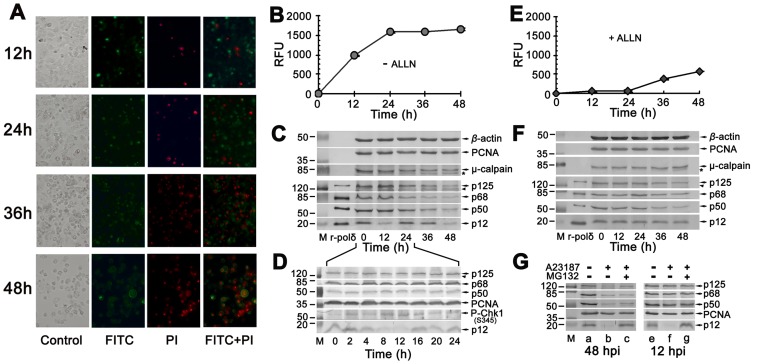
Degradation of p12 subunit in calcium ionophore A23187 treated HeLa cells. (**A**) Observation of apoptotic cells by Annexin V staining. The HeLa cells were treated with A23187 and the cells undergoing apoptosis were observed on a fluorescence microscope at indicated time shown on the left with Annexin V-FITC apoptosis detection kit as described in Materials and Methods. FITC: detection of PS in the early stage of apoptosis by FITC-conjugated Annexin V in green. PI: PI staining of nucleus in the later stage of apoptosis in red. FITC+PI: merger for FITC and PI. (**B**) Activation of calpain in apoptotic HeLa cells by the treatment with A23187 in the absence of inhibitor ALLN. The activities were measured with calpain activity fluorometric assay kit as described in Materials and Methods. The vertical axis indicates an increased calpain activity as RFU (relative fluorescent unit). The horizontal axis shows the time after treatment with A23187. (**C**) Degradation of p12 in apoptotic HeLa cells by the treatment as in panel (B) by Western blot analysis. The numbers shown on the left indicate the positions in kDa protein markers. The four subunits of Pol δ, PCNA, μ-calpain, and *β*-actin are indicated by arrows. The autoproteolytic cleavage products of μ-calpain are shown by an asterisk. While ∼118 kDa proteolytic cleavage products of p125 are indicated by arrowhead. M: protein marker in kDa. r-pol δ: highly purified recombinant human DNA Pol δ four-subunit complex as a control. (**D**) Degradation of p12 in apoptotic HeLa cells with shortened time points in every 4 hours. The treatment of cells was performed as in panel (C). The time points for Western blot analysis are indicated as 0, 2, 4, 8, 12, 16, 20, and 24 h. The detected phospho-Chk1 (S345) is also indicated by an arrow besides four subunits of Pol δ and PCNA. (**E, F**) Exact parallel assays as in panels (B, C) except for the treatment of HeLa cells in the presence of inhibitor ALLN. (**G**) Blockage of p12 proteolysis by inhibitor MG132. Western blot analyses were performed for blockage of p12 proteolysis by MG132 at 48 hpi (left panel) and 12 hpi (right panel). M: protein marker in kDa. a and e: non-treated HeLa cells as negative control. b and f: A23187 treated HeLa cells in the absence of inhibitor. c and g: A23187 treated HeLa cells in the presence of MG132.

Biochemical and genetic analysis of apoptosis has shown that intracellular proteases are key effectors in cell death pathways. For the observation of p12 response during the calcium-triggered apoptosis, the calcium ionophore A23187 was used to treat HeLa cells for the activation of calpain [19], [20]. As shown in Fig. 1A, the induced apoptotic cells could be easily observed by both Annexin V-FITC and Propidium Iodide (PI) staining, as described in Materials and Methods. After initiating apoptosis, cells translocate the membrane phosphatidylserine (PS) from the inner face of the plasma membrane to the cell surface. The PS was detected by staining with a fluorescent conjugate FITC of Annexin V in the plasma membrane showing green (Fig. 1A, FITC) while cells that have lost membrane integrity showed red (Fig. 1A, PI) throughout the nucleus by PI staining.

Following the treatment of A23187, the calpain activities were significantly stimulated at 12 hours post-induction (hpi) and reached a saturation levels after 24 hpi, by the measurement of calpain substrate Ac-LLY-AFC (Materials and Methods), as shown in Fig. 1B. In consistent with the activation of calpain, the autoproteolytic cleavage of the 80-kDa subunit to a 75-kDa fragment [20] was observed in Fig. 1C (the 80-kDa μ-calpains are indicated by an arrow and the 75-kDa cleavage products by an asterisk). The 75-kDa fragments of μ-calpain were observed at 24 hpi and increased in a time–dependent manner up to peak levels by 48 hpi. The p12 subunit was degraded at both 12 hpi and 48 hpi (Fig 1C, p12; Fig. 1G, f and b). The appearance of about 118-kDa fragments of Pol δ catalytic subunit p125, correlated with substantial activation of μ-calpain, was detected from 24 to 48 hpi by immunoblot as shown in Fig. 1C (the 125-kDa Pol δ catalytic subunits are indicated by an arrow and 118-kDa cleavage products by a filled triangle). The loss of sequences at the N- or C-termini of p125 is unknown. The levels of other two subunits of Pol δ, p68 and p50, were decreased also only at 48 hpi while PCNA was unaffected (Fig. 1C; Fig. 1G, b).

Interestingly, several repeats showed that the degradation of p12 occurred at 12 hpi then returned to normal p12 levels at 24–36 hpi. Finally, the depletion of p12 was observed again at 48 hpi (Fig 1C, p12). To explore this unusual phenomenon that the degradation of p12 was observed at 12 hpi then its levels turned back, we shortened the sample time points in every 4 hours in the first 24-hour period. As shown in Fig. 1D, a specific depletion of p12 subunit occurred as a time–dependent manner. The initiation of p12 depletion required 4 hours. The p12 levels were depleted below detectable levels by 12 hours and recovered by 16 hours thereafter. During the whole 24-hour period of testing, the other three subunits of Pol δ and PCNA were relatively unaffected. The immunoblot for Chk1-pS345 was also performed. Corresponding to the p12 depletion to near completion at 12 hpi, the phospho-Chk1 (S345) was detected (Fig. 1D, P-Chk-1 S345), implying that the degradation of p12 may relate to the ATR/Chk-1 signalling pathway in the calcium-triggered apoptosis during the early 24-hour period.

The HeLa cells were pre-incubated for 3 hours with 10 μM calpain inhibitor ALLN and then treated with 4 μM A23187. The calpain activities were efficiently inhibited in a time-dependent manner up to 36 hpi which confirmed that A23187 activates the activity of calpain. There still exist some of calpain activities at 48 hpi that might result from the insufficient amount of ALLN or some unknown mechanisms (Fig. 1E). Therefore, the μ-calpain autoproteolysis was still observed slightly at 48 hpi (Fig. 1F, the 75-kDa cleavage products are indicated by an asterisk). Correspondingly however, the depletion of p12 was efficiently blocked during the whole period of cell apoptosis (Fig. 1F, p12). It implies that the p12 degradation might be mediated by μ-calpain. The appearance of about 118-kDa fragments of p125 subunit of Pol δ was not observed (Fig. 1F, p125). Notably, inhibitor MG132 could also efficiently block the degradation of p12 subunit at both 12 hpi and 48 hpi (Fig. 1G), suggesting that the degradation of p12 might be not only by the contribution of μ-calpain but also through a proteasome pathway in calcium-triggered apoptosis. MG132 could not inhibit degradation of p125, p50, and p68 at 48 hpi (Fig. 1G, left panel).

### Cleavage of the smallest subunit p12 of Pol δ does not occur during apoptotic HeLa cells triggered by the effector caspases-3

**Figure 2:**
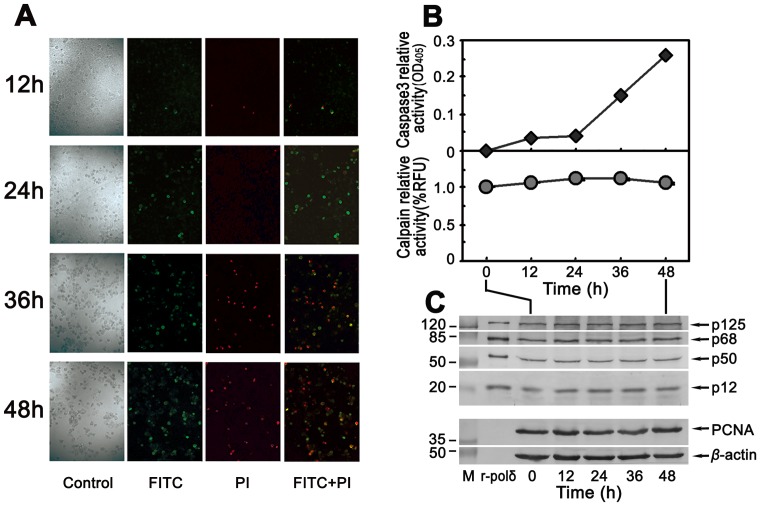
No degradation of p12 subunit in paclitaxel-cisplatin co-treated HeLa cells. (**A**) Observation of apoptotic cells by Annexin V staining. The HeLa cells were co-treated with paclitaxel and cisplatin as described in Materials and Methods and the cells undergoing apoptosis were observed on a fluorescence microscope as described for Fig. 1A. (**B**) Activation of caspase-3 in apoptotic HeLa cells by co-treatment with paclitaxel and cisplatin, but no activation of calpain. The caspase-3 activities were measured with caspase-3 activity assay kit as described in Materials and Methods (upper panel). The vertical axis indicates increased caspase-3 relative activities as OD_405_ (the absorbance at 405 nm). The horizontal axis shows the time after treatment. Parallel assays for calpain activities were performed with calpain activity fluorometric assay kit (lower panel). No obvious changes of calpain activities, shown as RFU%, were detected comparing with the control shown as “0” on the horizontal axis that indicates untreated cells. (**C**) No degradation of both p12 and other three subunits of Pol δ in apoptotic HeLa cells trigged by caspase dependent pathway. The effects on Pol δ subunits by co-treatment of HeLa cells with paclitaxel and cisplatin were analyzed by Western blot. M: protein marker in kDa. r-pol δ: highly purified recombinant human DNA Pol δ four-subunit complex as a control. The numbers from left to right indicate the time after treatment. The four subunits of Pol δ, PCNA, and *β*-actin are indicated by arrows.

To investigate whether the degradation of p12 occurred during a caspase triggered apoptosis, the HeLa cells were treated with a combination of 10 μg/ml paclitaxel and 8 μg/ml cisplatin. The apoptotic cells were efficiently induced by the observation under a fluorescence microscope by Annexin V-FITC and PI staining (Fig. 2A). As shown in Fig. 2B (upper panel), the caspase-3 was activated in a time-dependent manner from 0 to 24 hpi then significantly up to a maximum by 48 hpi by the measurement of caspase-3 substrate Ac-DEVD-*p*NA (Materials and Methods). However, no calpain activities were detected during the whole the period of undergoing apoptotic cells (Fig. 2B, lower panel). Corresponding to the testing time points, no degradation of p12, as well as other three subunits of Pol δ and PCNA, was observed by immunoblot analysis (Fig. 2C). It suggests that the degradation of p12 subunit only took place in the calcium triggered apoptosis, but not in a caspase-dependent pathway triggered apoptotic cell death.

### The subunit p12 is a substrate of μ-calpain, but not caspase-3, *in vitro*

**Figure 3:**
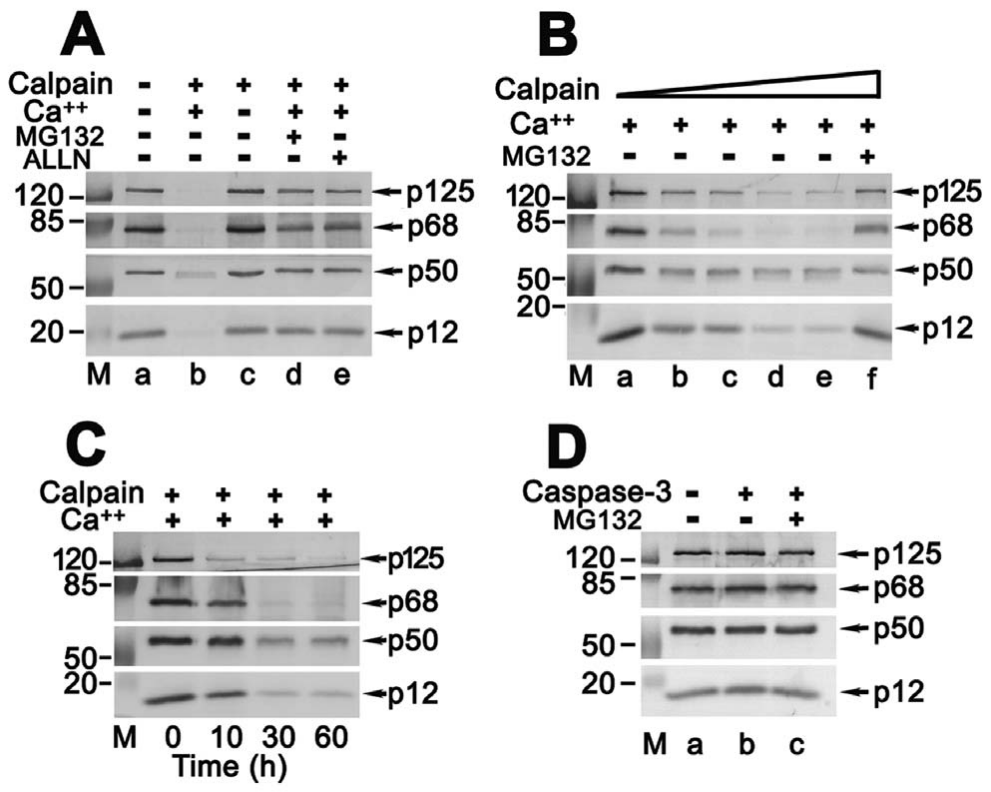
Cleavage of the p12 subunit by μ-calpain or caspase-3 *in vitro*. (**A**) About 480 ng recombinant heterotetramer was incubated with 1 unit of μ-calpain in a reaction buffer for 1 h at 30°C as described in Materials and Methods. The reactions were analyzed by Western blot. M: protein marker in kDa. a: Pol δ4 negative control. b, c: Pol δ4 incubated with μ-calpain in the presence or absence of 6 mM CaCl_2_. d, e: Pol δ4 incubated with μ-calpain in the presence of inhibitors MG132 or ALLN. Four subunits of Pol δ are marked by arrows. (**B**) Proteolysis of Pol δ4 complex by μ-calpain as a dose-dependent manner. The reconstituted cleavage assays were performed as described in Materials and Methods and the reactants were analyzed by Western blot. a-e from left to right indicates the increased units of μ-calpain as 0, 0.125, 0.25, 0.5, and 1 (units) in assays. f: same assay as in “e” except for the presence of inhibitor MG132. (**C**) Time-course of proteolysis of Pol δ4 complex by μ-calpain. The reconstituted cleavage assays were performed as in (A). The reactants were analyzed by Western blot at indicated time. (**D**) No proteolysis of Pol δ4 complex by caspase-3. About 480 ng recombinant heterotetramer was incubated with 1 unit of caspase-3 in a reaction buffer for 1 h at 30°C as described in Materials and Methods. The reactants were analyzed by Western blot. M: protein marker in kDa. a: Pol δ4 negative control. b, c: Pol δ4 incubated with caspase-3 in the absence or presence of inhibitor MG132.

The *in vivo* experiments indicate that the p12 was depredated only in the calcium triggered cell death. To directly evaluate if the p12 is a substrate of μ-calpain, a reconstituted cleavage assay was performed. Truly, not only p12, but also other three subunits of Pol δ were proteolysed by one unit of the active human μ-calpain in the presence of 6 mM of activator Ca^2+^ in the reaction (Fig. 3A, b). In the absence of Ca^2+^ or addition of inhibitor ALLN or MG132, the proteolysis of subunits of Pol δ was efficiently blocked (Fig. 3A, c, d, and e).

The subunits of Pol δ were proteolysed as a dose-dependent manner and 0.125 units of μ-calpain could initiate the degradation (Fig. 3B). Thirty minutes reaction time was enough to cleave them all (Fig. 3C). However, no any proteolysis of Pol δ subunits was observed by the active human caspase-3 (Fig. 3D). These results are consistent with the observations in apoptotic HeLa cells triggered by calpain activation or caspase-dependent pathway, as described above.

### Determination of the μ-calpain cleavage sites in p12 subunit

**Figure 4:**
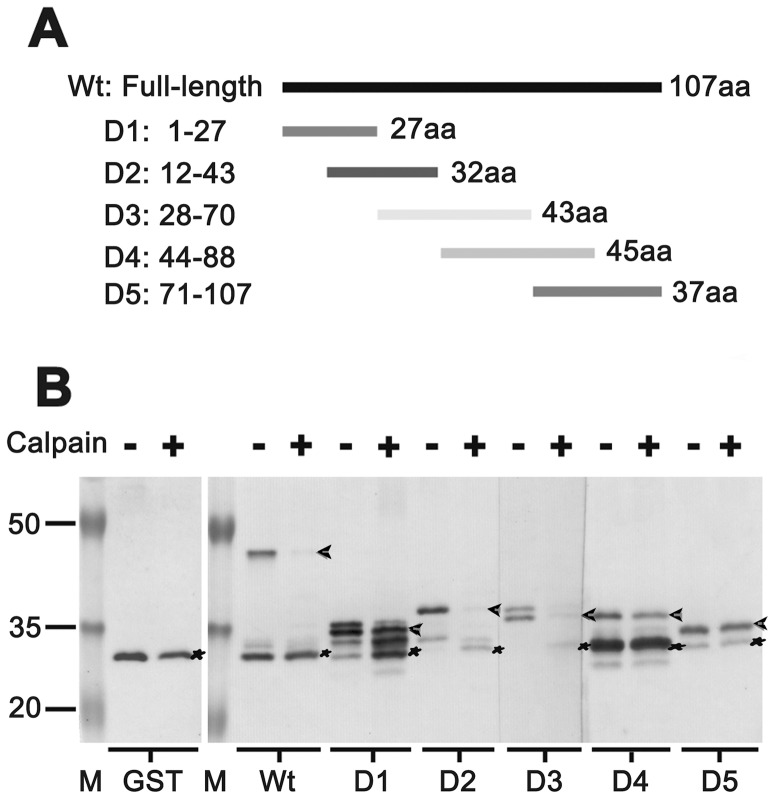
Determination of the μ-calpain cleavage sites in p12 subunit. A series of highly purified GST-tagged p12 full-length and deletions D1-D5, designed as in (A), were used for the determination of candidate substrates of μ-calpain. Each 0.5 μg of five purified GST-tagged p12 truncated fusion proteins was incubated with one unit of μ-calpain in the presence of 6 mM CaCl_2_ by the standard *in vitro* cleavage assays as described in Materials and Methods. The reactants were analyzed by Western blot (B). GST protein (marked as GST) was as the control. In each reaction, no μ-calpain adding was as a negative control. The positions of GST-p12 full-length (marked as Wt) or individual five deletions (marked as D1, D2, D3, D4, and D5) are indicated by arrows and GSTs are indicated by asterisks.

To estimate the cleavage sites by μ-calpain in p12 subunit, we generated a series of GST tagged p12 truncated fusion proteins, D1-D5, designed as shown in Fig. 4A that were expressed in *E. coli* and purified with glutathione sepharose 4B beads. The location of cleavage sites in p12 fragments D1-D5 was measured by the standard *in vitro* cleavage assays as described in Materials and Methods. The GST protein was used as a negative control while the GST tagged full-length p12 fusion protein was used as a positive control. As shown in Fig. 4B, the cleavage only occurred within D2 and D3. Analysis based on primary data suggests that the cleavage sites by μ-calpain located within the overlap between D2 and D3, *i.e*. ^28^LAPELGEEPQPRDEEE^43^.

### Determination of the interaction of μ-calpain with p12 as well as with other three subunits of Pol δ and PCNA

The elucidation of the mechanisms of substrate cleavage by calpains requires complex combinatorial analysis on binding sequences around substrate cleavage sites. Therefore, we examined the direct interactions of μ-calpain with the individual subunits of Pol δ and PCNA in pairs.

**Figure 5:**
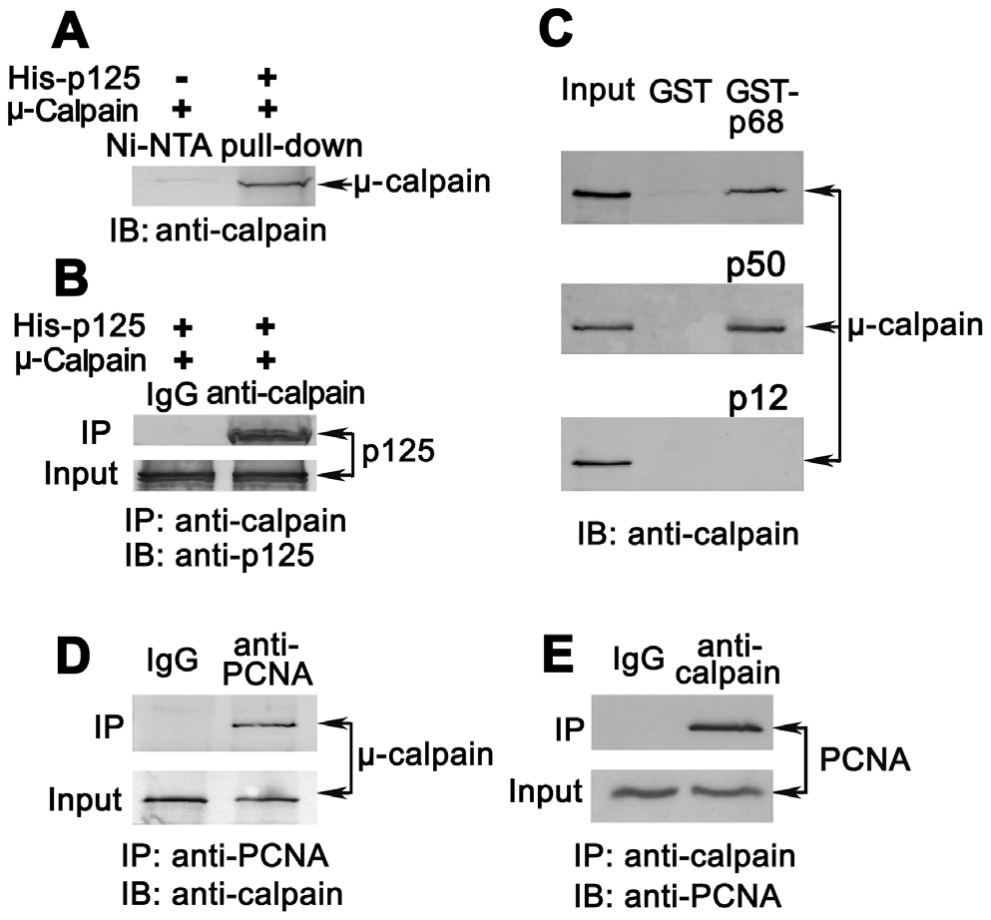
Determination of the interaction of human μ-calpain with individual Pol δ subunits and PCNA. (**A, B**) The interaction of catalytic subunit p125 with μ-calpain. Ni-NTA pull-down assays were first performed by the interaction of 0.5 μg His-tagged p125 and 0.5 μg μ-calpain as described in Materials and Methods. The pull-down productions by Ni-NTA beads were analyzed by Western blot with anti-μ-calpain antibody (A). The reaction without adding His-tagged p125 was used as a negative control. The detected μ-calpain that binds to p125 was indicated by an arrow. The Co-IP was further performed by the reaction of 0.5 μg His-tagged p125 and 0.5 μg μ-calpain in the presence of anti-μ-calpain antibody. The immunoprecipitates were analyzed by Western blot with anti-p125 antibody (B). Substitution of anti-μ-calpain antibody with normal rabbit IgG in the reaction was used as the control. One twentieth of His-p125 was loaded as input. (**C**) Interaction of μ-calpain with individual p68, p50, and p12 subunit. The GST pull-down assays were performed by the use of GST-tagged p68, p50, or p12 fusion proteins as the baits to pull down μ-calpain while GST protein used as a negative control. The captured products by glutathione-Sepharose 4B beads were analyzed by Western blot with anti-μ-calpain antibody. One twentieth of μ-calpain was used as input. The positions of captured μ-calpain were marked by arrows. (**D, E**) Interaction of μ-calpain with PCNA by Co-IP assays. Co-IP assays were performed by the incubation of full-length human PCNA and μ-calpain proteins in the presence of PC-10 anti-PCNA mouse monoclonal antibody. Replacement of PC-10 with normal mouse IgG was used as the control. The captured μ-calpain was immuneblotted with anti-μ-calpain antibody, marked by arrows (D). One twentieth of μ-calpain was loaded as input. A revised Co-IP was performed by the use of anti-μ-calpain antibody for Co-PI and PC-10 antibody for immunoblot (E). One twentieth of PCNA was loaded as input. Replacement of anti-μ-calpain antibody with normal rabbit IgG was used as the control. Captured PCNA was immunoblotted with PC-10 marked by arrows.

The interaction of μ-calpain with the catalytic subunit p125 was firstly examined by Ni-NTA pull-down assays. The highly purified His-p125 was used as the bait-proteins to pull down active full-length μ-calpain by use of Ni-NTA agarose beads that were pre-treated with 2% BSA to reduce the binding of non-specific proteins. As shown in Fig. 5A, the captured μ-calpain could be easily detected by immunoblot with anti-μ-calpain antibody. While in the control in which no His-p125 was added in the reaction, no any μ-calpain was detected. It suggests that μ-calpain is able to bind to p125 subunit, which was further confirmed by Co-IP assays. The His-p125 was incubated with μ-calpain together with anti-μ-calpain rabbit polyclonal antibody in the presence of protein A/G agarose beads that were pre-treated with 2% BSA. The captured p125 was detectable by immunoblot with anti-p125 antibody (Fig. 5B, right column). In the control in which the anti-μ-calpain antibody was replaced with the normal rabbit IgG, no any p125 could be detected (Fig. 5B, left column).

Next, we examined the contribution of μ-calpain to the binding of other three subunits of Pol δ by GST pull-down assays. The GST-tagged p68, p50, or p12 fusion proteins were used as the baits to pull down μ-calpain, respectively, using glutathione-Sepharose 4B beads. As shown in Fig. 5C, the captured μ-calpains were detectable by immunoblot with anti-μ-calpain antibody when GST-tagged p68 or p50 fusion proteins were used as the baits, while GST protein, used as a negative control, could not pull down any prey proteins. No binding between μ-calpain and p12 was observed in our experimental condition (Fig. 5C, lower panel). It implies that μ-calpain could bind to Pol δ complex through p125, p68, and p50 subunits but not p12.

Besides its role as a molecular sliding clamp for DNA synthesis at replication fork that functions as a processivity factor to stimulate Pol δ activity [21], PCNA also plays a central role in various DDR pathways [1], [12]. Therefore, we also examined the interaction of μ-calpain with PCNA by Co-IP assays. The full-length human PCNA and active human μ-calpain proteins were incubated with PC-10 anti-PCNA mouse monoclonal antibody in the presence of protein A/G agarose beads. The captured μ-calpain was detectable by immunoblot with anti-μ-calpain antibody. While in the control in which the PC-10 was replaced with the normal mouse IgG, no any captured μ-calpain was detected (Fig. 5D). And vice versa, the captured PCNA was detectable when the anti-μ-calpain antibody was used in Co-IP and the anti-PCNA antibody was used to blot PCNA. In this reaction, the replacement of anti-μ-calpain antibody with normal rabbit IgG was taken as the control (Fig. 5E). These results imply that the μ-calpain is able to interact with PCNA.

## Discussion

A number of recent studies have suggested that the degradation of p12 subunit of human DNA polymerase delta that results in an interconversion between Pol δ4 and Pol δ3 forms plays a significant role in response to replication stress or genotoxic agents triggered DNA damage as stated in Introduction. However, little has been done to investigate its degree of participation in any of the more common apoptosis.

Early studies have demonstrated that the initiation and execution of apoptosis rely on a complex network of caspase proteases as mediators to regulate proteolysis during apoptotic cell death. Caspases take a major responsibility in the demise of cells that have been triggered to undergo apoptosis. They can functionally be divided into two main groups, initiator and effector caspases. Upon the commitment of the cell to die, initiator caspases are firstly to be activated and responsible for cleavage and activation of the effector caspases-3, -6, and -7. In turn, these activated effctors act on specific cellular substrates [22], [23]. Many caspase substrates have been identified, such as nuclear proteins, poly(ADP-ribose) polymerase (PARP)-1 and retinoblastoma (RB); structural proteins of the nucleus and cytoskeleton, lamins and gelsolin [24], [25], [26], [27]. Caspase-3 can mediate the cleavage of DNA fragmentation factor results in chromatin condensation and DNA fragmentation during apoptosis [28], [29].

Recent evidences have accumulated that non-caspases, including calpains, cathepsins, granzymes, and the proteasome complex, also play critical roles in mediating and promoting cell death [17], [30]. Calpain is an intracellular cysteine protease. It is activated by intracellular Ca^2+^ and cleaves and activates caspase-12 and cyclindependent kinase 5 (cdk5) to mediate calcium-triggered cell death [17], [18]. The calpains are characterized as calpain I (μ-calpain) and calpain II (m-calpain). These two ubiquitously expressed isozymes are each composed of the catalytic 80-kDa subunit and regulatory 30-kDa subunit. Activation of μ- and m-type of calpains requires micro- and millimolar concentrations of calcium, respectively [31]. Although calpains are well known to play a role in a wide range of metabolic pathways through limited proteolysis of their substrates, the exact mechanisms of substrate recognition and cleavage by calpains are still relatively unclear. The positions of the cleavage sites in substrates are crucial to gain insight into how calpains modulate cellular functions through their substrate proteolysis [32]. If cleavage sites are determined, specific antibodies against the sites and inhibitors for specific substrate proteolysis can be designed to analyze proteolytic events by calpains under various conditions. Unlike caspases that cleave their substrates in a highly sequence-specific fashion with a near absolute requirement for an aspartic acid in the P1 position, for example, activated caspase-3 cleaves its substrates at a conserved DXXD motif [22], [23], [33], the substrate cleavage by calpains requires a wide range of amino acid sequences around substrate cleavage sites [34], [35], [36], [37].

Interestingly, both caspase-3 and calpain can mediate the cleavage of the human DNA Pol ε catalytic subunit p261 to produce a p140 fragment both *in vivo* and *in vitro* [33]. For human DNA Pol δ, the p68 and p12 subunits are readily proteolyzed by μ-calpain *in vitro* [16]. Therefore, those human replicative polymerases are believed to be involved in a process of apoptosis in some ways and must play a role with an unknown mechanism. Here, we first present the appearance of Pol δ proteolysis by μ-calpain in calcium-triggered apoptotic HeLa cells. The observation of p12 complete degradation at 12 hpi and restoration thereafter by 24 hpi then depletion again after 36 hpi by μ-calpain in a time-dependent manner (Fig. 1C and D) suggests a dual function of Pol δ by its interconversion between Pol δ4 and Pol δ3 that is involved in a novel apoptosis mechanism. The μ-calpain binds to p12 in the vicinity of its cleavage sites, but not p12 itself, by the interaction of μ-calpain with other three subunit p125, p68, and p50, as well as PCNA (Fig. 5), which may be through a Pol δ4/PCNA complex that formed to response to DNA damage during apoptosis. The p12 cleavage sites by μ-calpain are located within a 16-amino acids peptide, *i.e.* ^28^LAPELGEEPQPRDEEE^43^ (Fig. 4). Although it is generally accepted that calpains are involved in many cell death processes, their exact role is still unclear as it is not well characterized as that for the caspases. It is thought likely that calpain is activated by an initial insult via a rise in intracellular calcium from the endoplasmic reticulum (ER), the mitochondria or an influx of extracellular calcium [38]. Also, calpain might be activated through a substitutable kinase signalling pathway in the absence of cytosolic calcium fluxes [39], [40], [41]. Once activated, calpains degrade membrane, cytoplasmic and nuclear substrates, leading to the breakdown of cellular architecture and finally apoptosis [42].

**Figure 6:**
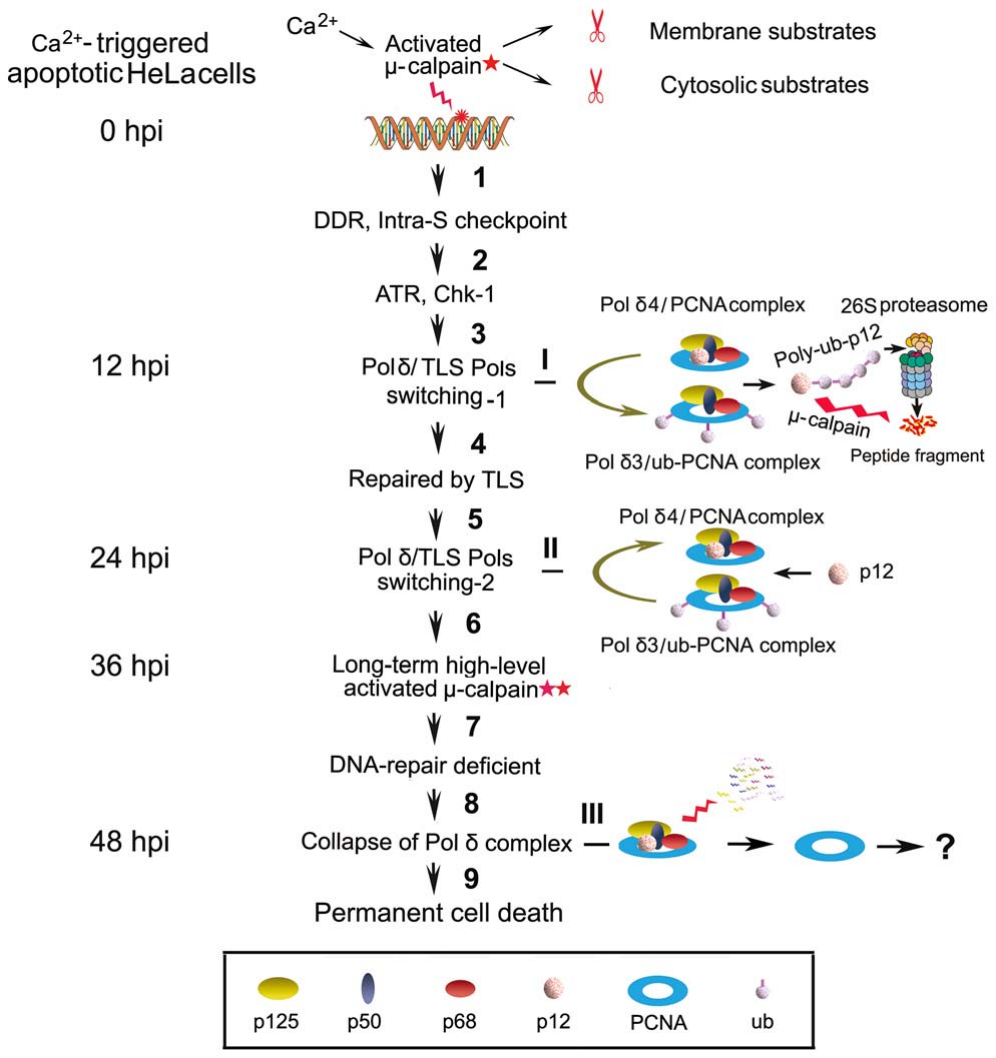
A proposed working model for the responses of p12 and Pol δ to the calcium-triggered apoptosis in HeLa cells. This diagram broadly outlines the process of p12 degradation and conversion between Pol δ4 and Pol δ3 in the early stage of apoptosis and final collapse of Pol δ4 complex in the late stage of apoptosis. Step 1: The A23187 treatment leads to DNA damage upon μ-calpain activated at lower level (pentagram in red). In S-phase cells, these foci also include replication forks stalled at CPDs. These base lesions activate the DDR and intra-S-phase checkpoint. Step 2: Because the p12 degradation is ATR-dependent, ATR is recruited to DNA-damage sites or stalled replication forks by the activation of its downstream Chk-1 kinase through the phosphorylation of Ser345 and Ser317. Step 3: Complete degradation of p12 that may be through an intact ubiquitination-proteasome system by its poly-ubiquitination or through μ-calpain proteolysis leads to the conversion of the Pol δ4 to Pol δ3 at 12 hpi (I). Pol η is then recruited to Pol δ3/ub-PCNA, *i.e.*, first enzyme switching. Step 4: Those bulky lesions are bypassed via TLS by Pol η. Step 5: Then, the restoration of p12 into Pol δ3 complex to reconstitute Pol δ4 at 24 hpi (II) leads to switching again between Pol δ4 and Pol η through Pol δ4/PCNA complex. Step 6–9: In the presence of long-term high level activated μ-calpain which is marked as double pentagrams in red, bulky DNA adducts, DNA cross-links and DNA double-strand breaks (DSBs), etc., could not be repaired by multiple forms of DNA repair pathways during 24 to 48 hpi, which leads to a total collapse of Pol δ complex (III), accompanying with the collapse of replication forks, chromatin condensation, and DNA fragmentation, finally cell permanent death.

Taking into account the earlier delineation of the major features of the degradation of p12 in response to DNA damage and combining with the observations in this work, we are able to propose a hypothesis that allows us to outline a probable process. The activated μ-calpain initials the degradation of p12 and converts the nuclear pool of Pol δ4 to Pol δ3 lacking p12 in response to calcium-triggered apoptosis in the early stage, thereafter leads to a complex degradation of Pol δ in the later stage, and consequently, collapse of replication fork, chromatin condensation and DNA fragmentation, finally apoptosis. In this hypothesis as shown in Fig. 6, upon μ-calpain activated at lower level, major lesions such as cyclobutane pyrimidine dimers (CPDs) and [6-4]pyrimidine–pyrimidone photoproducts (6-4PPs) are accumulated in damage foci, which activates the DDR and intra-S-phase checkpoint [1]. ATR, which activates its downstream Chk-1 kinase by the phosphorylation of Ser345 and Ser317, is recruited to DNA-damage sites or stalled replication forks. DDT pathway is activated, whereby translesion synthesis polymerases (TLS Pols) allow bypass synthesis across bulky lesions [13]. TLS is triggered by mono-ubiquitination of PCNA [43], which in turn functions to recruit Pol η in the case of DNA damage and subsequent switching between Pol δ and Pol η [12], [44]. It is suggested that Pol δ3 is the primary form of Pol δ that is available to participate in this switching [1], [14]. Previous studies show that the degradation of p12 is mainly through an intact ubiquitination-proteasome system by its poly-ubiquitination which is mediated by the E2/E3 pair, ubiquitin conjugating enzyme UbcH5c/RNF8 [14]. Here, Pol δ3 can also be formed through calpain-triggered p12 degradation pathway in the early stage of apoptosis. We cannot distinguish the exact mechanisms in this work because the inhibitor ALLN or MG132 can inhibit both calpain and proteasome activities. Probably, both pathways are involved in the early stage. Nevertheless, complete depletion of p12, which occurs at 12 hpi, leads to conversion of Pol δ4 to Pol δ3. Pol η is then recruited to Pol δ3/ub-PCNA via binding of its UBZ domain and performs bypass synthesis at primer terminus while Pol δ3 remains attached to ub-PCNA via the PIP-box located at the end of the C-terminus of p68 [1], a first enzyme switching between Pol δ3 and Pol η. After bulky lesions are bypassed via TLS by Pol η, a second enzyme switching may occur between Pol η and Pol δ through the Pol δ4/PCNA complex by the re-entry of p12 into the Pol δ3 complex to reconstitute Pol δ4 at 24 hpi (Fig. 1, C and D). However, in the presence of long-term high level activated μ-calpain, bulky DNA adducts, DNA cross-links and DNA double-strand breaks (DSBs), etc., could not be repaired by multiple forms of DNA repair pathways during 24 to 48 hpi, which leads to a total degradation of Pol δ complex (Fig. 1, C and G left panel), accompanying with the collapse of replication forks, chromatin condensation, and DNA fragmentation, finally cell permanent death. Thus, Pol δ is a target as a nuclear substrate of calpain in calcium-triggered apoptosis.

There are still many unanswered questions that need to be addressed. The large catalytic subunit p125 of Pol δ was cleaved into an approximate 118-kDa fragment that was recognized by the antibody ZJR12501 in calcium-triggered apoptotic HeLa cells (Fig. 1C). Do the losses occur at its N- or C-termini? What is the function of nicked 118-kDa fragment vis-a'-vis apoptosis and how is it integrated into Pol δ4 (δ4)/ub-PCNA complex? Also, there is still some uncertainty on interpreting the degradation of p12 through an ubiquitin-proteasome pathway or by the cleavage by calpain during the early stage of apoptosis. Moreover, our *in vitro* cleavage assays show that all four subunits of Pol δ were proteolyzed by μ-calpain, which is not very consistent with the previous observation in which only two subunits, p68 and p12 were readily degraded by μ-calpain while the p125 and p50 subunits were resistant [16]. It could be explained that the highly purified recombinant human DNA Pol δ four-subunit complex used in cleavage *in vitro* assay is from the hemolymph of infected silkworm larvae, which might be more close to physiological conditions that is further confirmed by our *in vivo* experiments (Fig. 1, C and G). With regard to the cleavage sites in p12 by μ-calpain, we have difficulty to locate the exact cleavage sites in p12 because the substrate cleavage sites by calpain are based on more complicated complex enzymatic reactions, not like caspases. Our experiments imply that the proteolysis of p12 is through the binding of μ-calpain to other three subunits of Pol δ and PCNA (Fig. 5) in the vicinity of p12. Probable cleavage sites were within a 16-amino-acid-residue fragment 28–43 (Fig. 4). It is believed that the involvement of calpains in apoptosis is limited to certain cell types and to specific stimuli [45], [46]. Therefore, if Pol δ could be considered as a marker for a nuclear substrate of calpain in Ca^2+^-triggered signal transduction pathways, it needs to be evaluated in more cell lines.

Intracellular Ca^2+^ overload and calpain activation are thought to be responsible for the induction of many neuronal diseases such as cerebral ischemia, Alzheimer's, Parkinson's, Huntingtons disease, and amyotrophic lateral sclerosis [47], and associate with a variety of human tumours such as colon cancer and colorectal adenocarcinoma [48]. It is believed that the chromosomal DNA is the main target of most cytotoxic anticancer drugs that react either directly with DNA through reactive metabolites or indirectly through the incorporation into DNA, or by blockade of DNA-metabolizing functions such as DNA polymerases or topoisomerases [49], [50], [51]. Therefore, our findings in this work are highlighted by the fact that the response of p12 to Ca^2+^-trigged apoptosis. The p12/Pol δ appears to be a potential marker in the study of the chemotherapy of cancer therapies. The assessment of effectiveness and reliability of those anticancer drugs could combine with a complex enzymatic determination of Pol δ levels based on the fact that all four subunits of Pol δ are depleted in the later stage of Ca^2+^-trigged cell death.

## Materials and Methods

### Cell culture and treatments

HeLa cells were maintained in Dulbecco's modified Eagle's medium (DMEM) with 10% heat-inactivated fetal bovine serum (FBS) in 5% CO_2_ in a humidified environment at 37°C as previously described [7], [15]. In most assays, the 100-mm culture dishes were seeded with 1×10^6^ cells in 5 ml of growth medium and allowed them to expand to about 85% to 90% confluence. Cell treatments were performed with the following compounds: Ca^2+^ ionophore A23187 (Sigma), paclitaxel and cisplatin (BioVision), and inhibitor ALLN or MG132 (BioVision).

### Detection of cell apoptosis by annexin V staining

HeLa cells cultured in 24-well plates were treated with 4 μM Ca^2+^ ionophore A23187 or with paclitaxel (10 μg/ml) and cisplatin (8 μg/ml). Cells undergoing apoptosis at the indicated time were identified using Annexin V-FITC apoptosis detection kit (Abcam), following the manufacturer's instructions. The cells were visualized and imaged on an Axiovert 200 M fluorescence microscope (Zeiss).

### Calpain activity assay

The calpain activities were measured using calpain activity fluorometric assay kit (BioVision), according to the manufacturer's instructions. Briefly, collected cells with different treatments at different time were extracted with an extraction buffer. The extracted cytosolic proteins without contaminations of cell membrane and lysosome proteases were incubated with the reaction buffer and calpain substrate Ac-LLY-AFC at 37°C for 1 hour in dark. Cleavage of a substrate was quantified in a fluorometer equipped with a 400 nm excitation filter and 505 nm emission filter. The untreated cells were taken as negative control. The activity was expressed as relative fluorescent units (RFU).

### Caspase-3 activity assay

Relative caspase-3 activities were determined using the caspase-3 activity assay kit (Beyotime Institute of Biotechnology, Haimen, China) as described [52]. Following the manufacturer's protocol, the cell lysates were incubated with reaction buffer and caspase-3 substrate Ac-DEVD-*p*NA for 2 hours at 37°C. Cleavage of a substrate was quantified by measuring the absorbance at 405 nm. One unit is the amount of enzyme that will cleave 1 nmol of the colorimetric substrate Ac-DEVD-*p*NA per hour at 37°C under saturated concentrations.

### Proteins and antibodies

Active human μ-calpain (calpain I) and active recombinant human caspase-3 were purchased from BioVision Incorporated. Recombinant human PCNA was over expressed in *E. coli* DH5-α and purified as previously described [2], [53]. Recombinant human DNA Pol δ four-subunit complex was purified from the hemolymph of infected silkworm larvae as previously described [2]. His-tagged p125, expressed in Sf-9 cells using the pFastBac HT vectors in the Bac-to-Bac system (Invitrogen) according to the manufacturer's instruction, was purified on Ni-NTA beads (Qiagen) and further purified by FPLC chromatography on a Mono-Q anion exchange column as previously described [16]. GST tagged full-length p68, p50, and p12 in the pGEX-5X-3 vector (GE Healthcare) were expressed in *E. coli* BL21DE3(plys), purified on glutathione beads (Amersham Biosciences), and further purified by FPLC chromatography on a Mono-Q anion exchange column as previously described [16], [54].

Anti-phospho-Chk1 (Ser345) rabbit polyclonal antibody and calpain I large subunit (μ-type) rabbit polyclonal antibody were purchased from Cell Signalling Technology. PC-10 mouse monoclonal anti-PCNA was purchased from Santa Cruz Biotechnology. The antibodies for Pol δ subunits were: mouse polyclonal antibody against p50 (ZJM5002), rabbit polyclonal antibodies against p125 (ZJR12501), p68 (ZJR6803), and p12 (ZJR1204), respectively, as described previously [2], [53].

### *In vivo* induction of p12 cleavage in HeLa cells

The activation of calpain and cell apoptosis was induced by the addition of 4 μM Ca^2+^ ionophore A23187. Three hours before A23187 treatment, the calpain I inhibitor ALLN (10 μM) or proteasome inhibitor MG132 (10 μM) was added to block the activation of calpain. The cell apoptosis and activation of caspase were induced by the addition of 10 μg/ml paclitaxel and 8 μg/ml cisplatin. The treated HeLa cells cultured in 100-mm dishes were harvested at indicated time and the pellets were re-suspended with an equal volume of cell extraction buffer (20 mM Tris-HCl, pH 7.8, 10% glycerol, 0.5 mM EGTA, 1 mM EDTA, 1 mM MgCl_2_, 200 mM NaCl, 0.1% NP-40, 1 mM phenylmethylsulfonate, 2 mM DTT, and protease inhibitor mixture). After 30 min of incubation on ice, cells were lysed by gently passing through a 23 G needle ten times and centrifuged at 12,000 rpm for 5 min. The resultant supernatants (total cell lysate) were used for the analysis of p12 subunit proteolysis by Western blot. Non-treated cells were taken as a control. The total protein concentration was measured with the Bradford assay.

### *In vitro* recombinant Pol δ four-subunit complex cleavage assay by human μ-calpain or recombinant human caspase-3

Standard cleavage reaction mixture in a total volume of 20 μl containing 480 ng recombinant heterotetramer in a reaction buffer (40 mM Tris-Cl, pH7.5, 120 mM NaCl) was incubated in the presence of 1 unit of human μ-calpain or caspase-3. Incubations were at 30°C for 1 hour in the presence of activator (6 mM CaCl_2_) only for calpain or different inhibitors (26 μM ALLN or 21 μM MG132). The generated products were separated by 12.5% SDS-PAGE gel and transferred to nitrocellulose membrane for Western blot analysis.

### Determination of the μ-calpain cleavage sites in p12 subunit

The full-length cDNA *POLD4* coding for p12 [55], kindly provided by Dr. Ellen H. Fanning, was used as a template for PCR to generate a series of p12 truncations. The PCR constructs were subcloned and inserted into pGEX-5X-3 vector between *Bam*HI and *Eco*RI sides to produce GST-tagged p12 truncated fusion proteins according to manufacturer's instruction (GE Healthcare). The primer pairs were as follows (the *Bam*HI and *Eco*RI sides are underlined, respectively):

D1, p12_1-27_, forward (5′-CGCGGATCCGCATGGGCCGGAAGCGGCTCATC-3′) and revise (5′-CCGGAATTCTCACTCCCCCTTGCTGTGCCCAGC-3′);

D2, p12_12-43_, forward (5′-CGCGGATCCGCCCGGTTGTGAAGAGGAGGGAG-3′) and revise (5′-CCGGAATTCTCATTCCTCCTCGTCGCGGGGCTG-3′);

D3, p12_28-70_, forward (5′-CGCGGATCCGCCTGGCACCCGAGCTAGGGGAG-3′) and revise (5′-CCGGAATTCTCACCAGCGCTGCAGCCGTGTGAT-3′);

D4, p12_44-88_, forward (5′-CGCGGATCCGCGCGGAGCTGGAGCTGCTGAGG-3′) and revise (5′-CCGGAATTCTCACAGCACCTGCCACACCTCTGG-3′);

D5, p12_71-107_, forward (5′-CGCGGATCCGCTGTCGGGCCAAGCATATGGGC-3′) and revise (5′-CCGGAATTCTCATAGGGGATAGAGATGCCAGAG-3′).

All generated constructs were verified by DNA sequencing and a series of GST-tagged p12 truncations were expressed in *E. coli* BL21DE3 (plys) and purified on glutathione beads. For the establishment of p12 sites which are substrate for μ-calpain, 0.5 μg each of five purified GST-tagged p12 truncated fusion proteins (D1–D5) was reacted with one unit μ-calpain in the presence of 6 mM CaCl_2_ by the standard *in vitro* cleavage assays. GST protein was as the control. The reaction products were resolved by 12% SDS-PAGE gel and transferred to nitrocellulose membrane for Western blot analysis with anti-GST antibody.

### Determination of the interaction of human μ-calpain with Pol δ individual subunits and PCNA

The interactions of μ-calpain with individual subunit p68, p50, and p12 were performed by GST tagged individual subunits pull down μ-calpain as previously described [54]. The interaction of catalytic subunit p125 with μ-calpain was determined by Ni-NTA pull-down assay. The 0.5 μg His-tagged p125 was incubated with 0.5 μg μ-calpain in 500 μl binding buffer (50 mM NaH_2_PO_4_, 300 mM NaCl, 20 mM Imidazole, 10% glycerol, pH 8.0) for 1 hour by end to end rotation. 15 μl of packed Nickel beads, pre-treated with 2% bovine serum albumin (BSA), were added and further rotated for 1 hour. The beads were spun down at 2,500 rpm for 5 minutes, washed 4 times with binding buffer, and followed by suspension in 2×SDS loading buffer and spinning down for 30 seconds at 14,000 rpm. The supernatant was loaded on SDS-PAGE, followed by transfer to nitrocellulose membrane and blot with anti-μ-calpain. The reaction without adding His-tagged p125 was used as a negative control. The interaction of p125 with μ-calpain was further confirmed by co-immunoprecipitation (Co-IP). 0.5 μg His-tagged p125 was incubated with 0.5 μg μ-calpain in reaction buffer (1×PBS, 1% NP-40, and protease inhibitor cocktail) for 1 hour by end to end rotation. Anti-μ-calpain antibody was added for 1 hour, followed by the addition of protein A/G agarose beads (Santa Cruz Biotechnologies) that were pre-treated with 2% BSA for 1 hour. The beads were spun down and washed 4 times with reaction buffer, followed by suspension in 2×SDS loading buffer and boiling for 5 minutes. The immunoprecipitates were separated by SDS-PAGE, followed by transfer to nitrocellulose membrane and blot with anti-p125 antibody. Substitution of anti-μ-calpain antibody with normal rabbit IgG (Santa Cruz Biotechnologies) in the reaction was used as the control.

The interaction of μ-calpain with PCNA was determined by Co-IP. Similarly, as described above, 0.5 μg PCNA and 0.5 μg μ-calpain were incubated with PC-10 anti-PCNA mouse monoclonal antibody. The immunoprecipitates were analyzed by immunoblotting with anti-μ-calpain antibody. The replacement of PC-10 with normal mouse IgG in the reaction was used as the control. Alternatively, the anti-μ-calpain antibody was used in Co-IP, and anti-PCNA antibody was used to blot PCNA. In this reaction, replacement of anti-μ-calpain antibody with normal rabbit IgG was taken as the control.

### Western blot analysis (Immunoblot, IB)

The total cell lysates of treated HeLa cells were separated by electrophoresis on 12.5% SDS-PAGE and transferred onto a nitrocellulose membrane. The membrane was then stained with Ponceau S and cut into several pieces according to the molecular weight of each designed proteins. The pieces of membrane were blocked with 5% w/v nonfat dry milk in TBST buffer (20 mM TrisHCl, pH 7.4, 150 mM NaCl, 0.05% Tween-20) for 1 hour at room temperature. The blots were then incubated with individual primary antibodys corresponding to each designed proteins for 1 hour at room temperature or overnight at 4°C. After three 15-min washes in TBST, the blots were incubated with AP-conjugated goat anti-mouse or anti-rabbit IgG (Pierce) for 1 hour and washed with TBST 3 times for 10 min. Perfect Protein Western Blot Kit (Novagen) was used for signal generation.
